# Prediction of HIF-1α expression in endometrial carcinoma by enhanced T_2_^∗^ weighted angiography and dynamic contrast-enhanced magnetic resonance imaging

**DOI:** 10.3389/fonc.2024.1439229

**Published:** 2024-12-11

**Authors:** Zongyuan Xie, Liangjie Lin, Changjun Ma, Ailian Liu

**Affiliations:** ^1^ Department of Radiology, The First Affiliated Hospital of Dalian Medical University, Dalian, China; ^2^ Department of MRI, North China University of Science and Technology Affiliated Hospital, Tangshan, China; ^3^ Clinical and Technical Support, Philips Healthcare, Beijing, China; ^4^ School of Biomedical Engineering, Faculty of Medicine, Dalian University of Technology, Dalian, China

**Keywords:** endometrial carcinoma, hypoxia-inducible factor-1α, enhanced T_2_^∗^ weighted angiography, dynamic contrast-enhanced, magnetic resonance imaging

## Abstract

**Purpose:**

To explore the value of quantitative imaging parameters by enhanced T_2_^*^ weighted angiography (ESWAN) and dynamic contrast-enhanced magnetic resonance imaging (DCE-MRI) for evaluating the expression of Hypoxia-inducible factor-1α (HIF-1α) in endometrial carcinoma (EC).

**Methods:**

Data from 122 patients with EC confirmed by clinical pathology were retrospectively analyzed. According to the number of positive cells stained with HIF-1α by immunohistochemistry, patients were divided into two groups: 65 cases with high expression of HIF-1α and 57 cases with low expression of HIF-1α. Clinical data included age, FIGO stage, menopausal status, abnormal uterine bleeding, and pathological type. All patients underwent preoperative 1.5T MRI scans, including ESWAN and DCE-MRI. The amplitude, phase, and R_2_^*^ values derived from ESWAN and the volume transfer constant (K^trans^), rate constant (K_ep_), and extravascular volume fraction (V_e_) values derived from DCE-MRI were measured by two observers, respectively. The intra-class correlation coefficient (ICC) was used to assess the measurement of reproducibility across observers, and the differences in imaging parameters between the two groups were compared using the independent sample t-test or Mann-Whitney U-test. Binary logistic regression analysis was used to find independent risk factors for HIF-1α expression. The efficacy of selected imaging parameters for predicting HIF-1α expression was assessed using receiver operating characteristic (ROC) curves, and the Delong test was used to compare the area under ROC curves (AUC).

**Results:**

The consistency between the two observers was good (ICC>0.75). The R_2_^*^, K^trans^, and K_ep_ values of the HIF-1α high expression group were higher than those of the HIF-1α low expression group (14.59 ± 4.06 *vs*. 11.99 ± 2.84 Hz, 0.45 ± 0.18 *vs*. 0.36 ± 0.14/min, and 2.17 ± 1.10 *vs*. 1.54 ± 0.80/min) (*P*< 0.001, *P* = 0.011, and *P* =0.001). Binary logistic regression analysis revealed that R_2_^*^ and K_ep_ values were independent risk factors for HIF-1α expression. The AUC values of R_2_^*^, K_ep_, and their combination for prediction of HIF-1α expression were 0.697, 0.677, and 0.781, respectively. The diagnostic efficacy was significantly improved with combination of R_2_^*^ and K_ep_.

**Conclusions:**

Quantitative parameters by ESWAN and DCE-MRI showed significant differences between EC patients with low and high expression of HIF-1α, and the combination of ESWAN and DCE-MRI improves the efficacy in prediction of HIF-1α expression in EC, which has an excellent clinical application prospect.

## Introduction

Endometrial carcinoma (EC) is the predominant gynecologic cancer in developed countries, exhibiting an escalating incidence globally (1). Due to the high mortality and recurrence rate, several studies have focused on evaluating the relationship between internal microenvironmental state of the tumor and clinical outcome of the patients (2, 3). Oxygen is vital in energy metabolism, tumors typically thrive in a hypoxic microenvironment. Malignant tumor cells, with their high energy needs, undergo metabolic reprogramming in this hypoxic setting. This promotes the survival of tumor cells, suppresses anti-tumor immunity, and promotes the growth of malignant tumors. Hypoxia-inducible factor-1α (HIF-1α) is the primary transcription factor regulating gene expression under hypoxia conditions (4). Previous studies have demonstrated a close relationship between HIF-1α expression and the oxygenation status of tumors, establishing it as a crucial biomarker of tumor hypoxia, invasiveness, or radioresistance (5, 6). The high expression of HIF-1α leads to high invasiveness or poor prognosis of EC. HIF-1α expression is usually detected through immunohistochemical staining of postoperative pathology. Obtaining the expression information of HIF-1α before treatment is helpful to quantitatively evaluate the degree of hypoxia of the tumor, thus helping to predict the treatment response or adjust the treatment plan as soon as possible.

Magnetic resonance imaging (MRI) techniques, especially several noninvasive and quantitative imaging methods, have been used to evaluate the hypoxia state in the human body before the operation, such as blood oxygen level-dependent (BOLD), diffusion-weighted imaging (DWI), intra-voxel incoherent motion (IVIM), and dynamic contrast-enhanced magnetic resonance imaging (DCE-MRI). BOLD reflects the changes of paramagnetic deoxyhemoglobin within erythrocytes and can be used to evaluate tumor oxygenation levels (7, 8). Given the generation of BOLD contrast is relatively cumbersome, its clinical application for tumor evaluation is limited. DWI and IVIM indirectly reflect tumor oxygenation status by assessing cell number levels in hypoxia. Li et al. (9) reported that the D value of the IVIM imaging was positively correlated with HIF expression, while the contrary results were found in another study (10).

The enhanced T_2_^*^ weighted angiography (ESWAN), based on the difference of magnetic sensitivity between different tissues and the effect of blood oxygen level dependence, can accurately measure iron deposition in living tissues (11, 12). Some studies have found a correlation between R_2_^*^ values obtained by ESWAN and hypoxia in rat glioma models (13). DCE-MRI could reflect the hemodynamic information of contrast agents in and out of the tumor, providing a quantitative reflection of tissue perfusion and an *in vivo* evaluation of tumor microcirculation status (14). Some studies have found that its quantitative parameters were related to hypoxia of soft tissue sarcoma (15) and nasopharyngeal carcinoma (16). This study aimed to preliminarily investigate the value of multiple quantitative parameters obtained by ESWAN and DCE-MRI in evaluating EC HIF-1α expression level.

## Materials and methods

### Research objects

**Figure 1:**
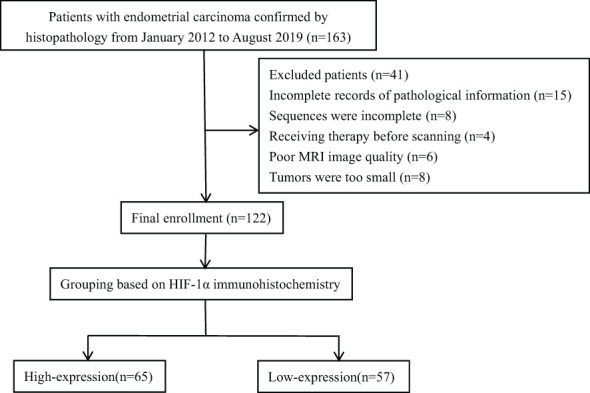
The study flow chart of patient enrollment.

The retrospective analysis encompassed the clinical and imaging data of EC in our hospital from January 2012 to August 2019. This research plan was approved by the hospital institutional review board, and all patients obtained written informed consent. Inclusion criteria (1): Patients confirmed by pathology to have EC with complete pathological data (2); MRI sequences included ESWAN and DCE-MRI (3); The largest diameter of the lesion was > 1 cm. Exclusion criteria (1): Patients who had received chemoradiotherapy or other treatments (including biopsy or curettage) before the MRI examination (2); Poor MRI image quality with artifacts that affected data measurement. The final enrollment included 122 EC patients. According to the proportion of HIF-1α positive cells, the patients were divided into two groups, including 65 cases in HIF-1α high expression group and 57 cases in HIF-1α low expression group. The research flow chart was shown in **Figure 1**.

### MRI imaging

MRI examinations were performed with a 1.5T MRI scanner (GE 1.5 T Signa HDXT MR) equipped with an 8-channel body coil within two weeks before operation. All patients were asked to urinate and fast for 4 to 6 hours before MRI examination. MRI sequences included T_1_WI, T_2_WI, DWI, ESWAN, and DCE-MRI. The DCE-MRI was collected for 30 phases, with each phase lasting 6 seconds. After the first phase of dynamic scans, the contrast agent Omniscan (Gd-DTPA-BMA, Nycomed Pharma, Norway) was intravenously injected at a concentration of 0.1mmol/kg of body weight, at a rate of 3 ml/s, followed by a 20 ml saline flush. The main scanning parameters were summarized in **Table 1**.

**Table 1:** The parameters of MRI scans.

Sequences	TR/TE (ms)	FOV (cm^2^)	Matrix (mm^2^)	Thickness/ gap (mm)	NEX	Scan time
Axial T_1_WI	680/10	30×30	320×192	1.0/1.0	2.0	1min and 37s
Axial T_2_WI	5660/88.4	30×30	288×224	5.0/1.0	3.0	3min and 13s
Axial DWI	3725/71.1	30×30	128×128	5.0/1.0	6.0	1min and 5s
Sagittal T_2_WI	3980/91.8	30×30	256×224	5.0/1.0	3.0	2min and 55s
Sagittal DWI	3725/71.1	31×31	192×192	5.0/1.0	6.0	1min and 15s
Axial ESWAN	16.5/5.1	40×40	256×192	2.0/0.8	5.0	21s
Sagittal DCE-MRI	3.2/1.5	35×28	256×192	3.6/0.0	0.69	3min and 30s

TR/TE, repetition time/echo time; FOV, field of view; NEX, number of excitations; DCE-MRI, dynamic contrast-enhanced magnetic resonance imaging; DWI, diffusion-weighted imaging.

### Imaging analysis

**Figure 2:**
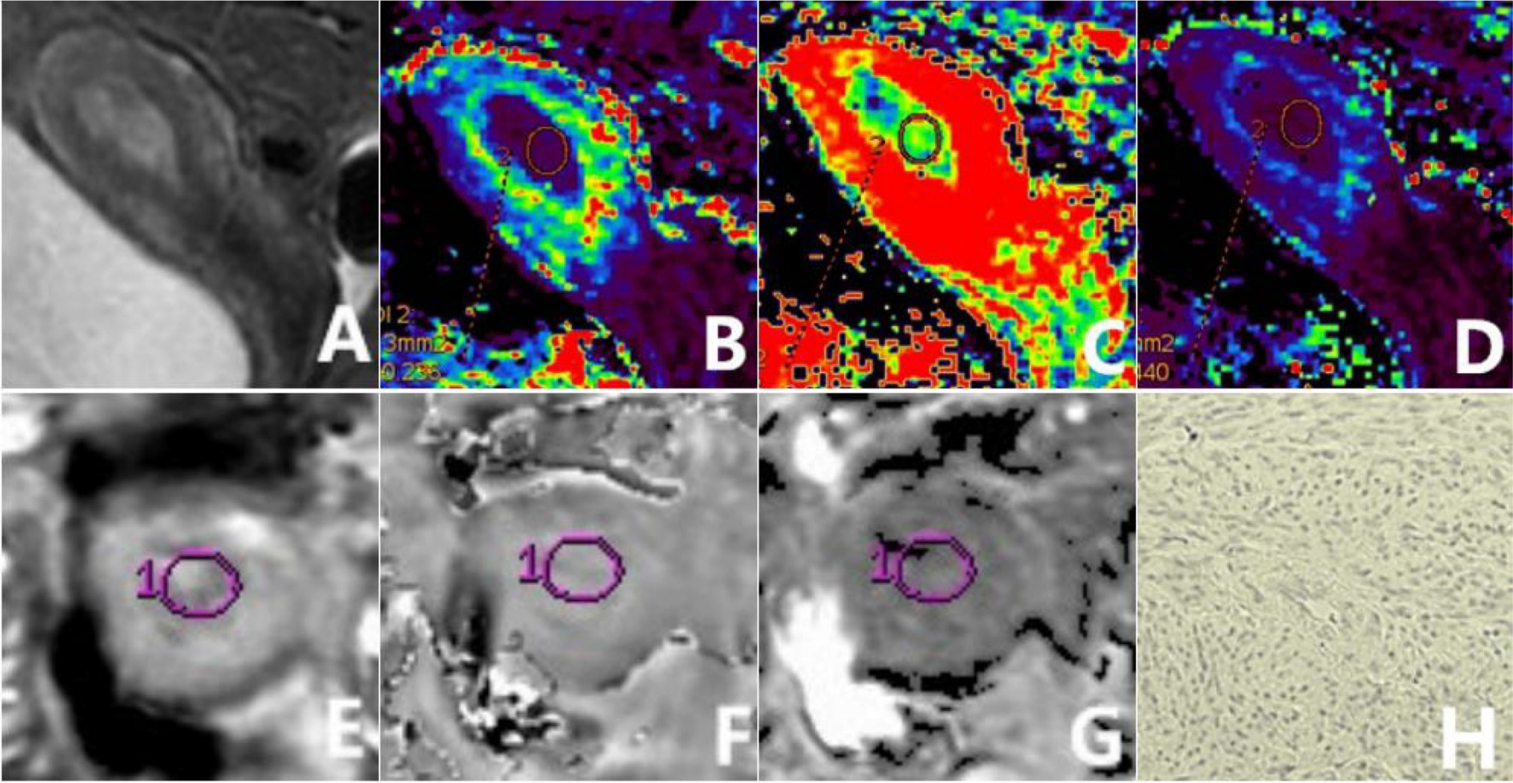
a 56-year-old patient diagnosed with type II EC of a poorly differentiated nature, exhibiting low HIF-1α expression. The sagittal T_2_WI **(A)** elucidates a pervasive, marginally elevated signal intensity within the uterine cavity, accompanied by a discontinuous signal in the junctional zone. The K^trans^ **(B)**, V_e_ **(C)**, and K_ep_ **(D)** maps of the sagittal DCE-MRI show quantitative values of 0.216 min^-1^, 0.302, and 0.753 min^-1^, respectively, for the tumor. The amplitude **(E)**, phase **(F)**, and R_2_^*^ **(G)** maps of the axial ESWAN show quantitative values of 953.13, 0.087 radians, and 13.842 Hz, respectively, for the tumor. The immunohistochemical staining image **(H)** (×200) reveals that HIF-1α staining positive cells is less than 50% and the staining intensity is less than 2 points.

**Figure 3:**
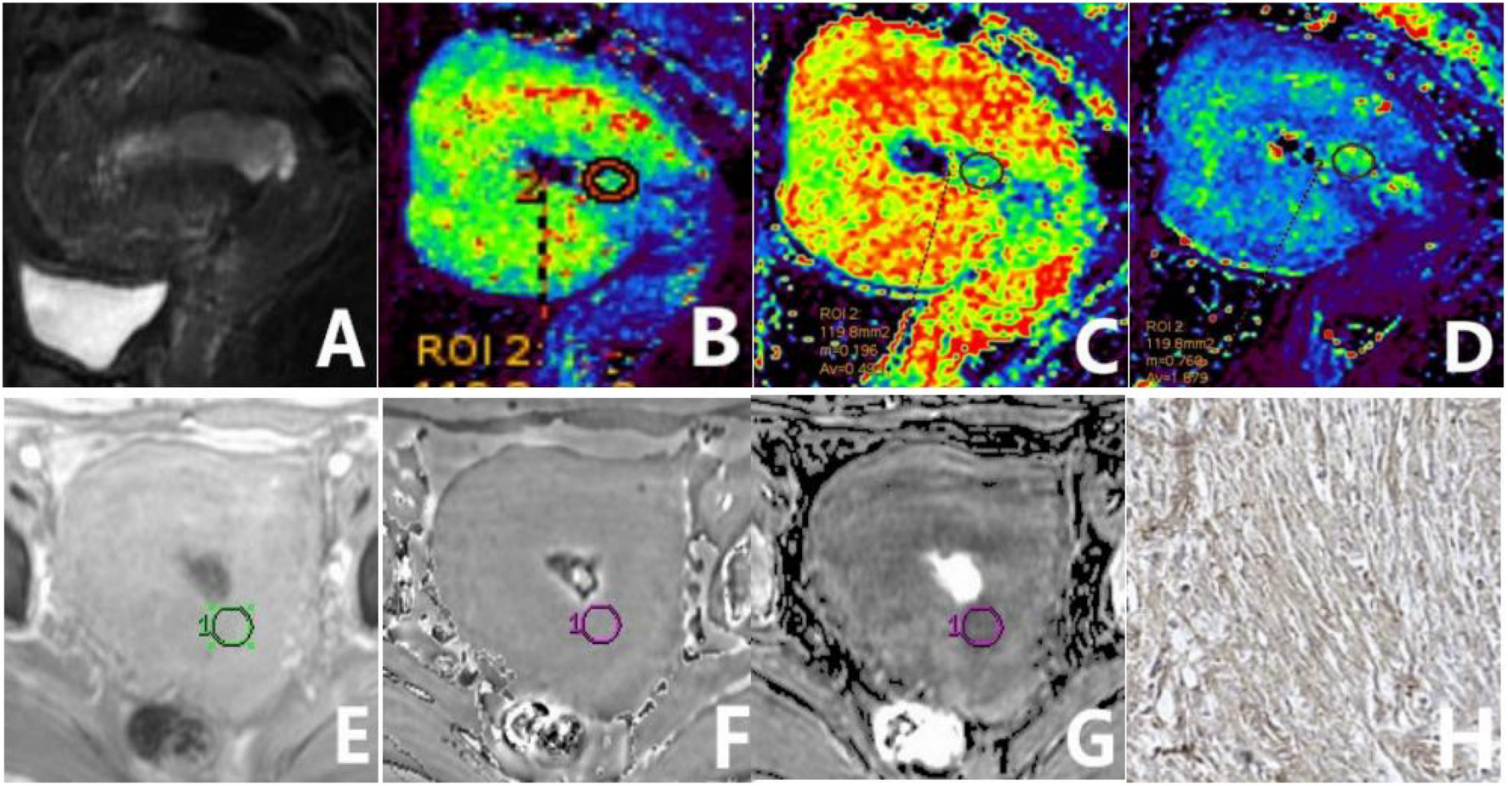
a 65-year-old patient diagnosed with type I EC of a highly differentiated nature, exhibiting high HIF-1α expression. The sagittal T_2_WI **(A)** elucidates a pervasive, marginally elevated signal intensity in the uterine cavity extending to the cervix, with an incomplete signal in the junctional zone. The K^trans^ **(B)**, V_e_ **(C)**, and K_ep_ **(D)** maps of the sagittal DCE-MRI show quantitative values of 0.547 min^-1^, 0.231, and 2.607 min^-1^, respectively, for the tumor. The amplitude **(E)**, phase **(F)**, and R_2_^*^ **(G)** maps of the axial ESWAN show quantitative values of 1370.4, 0.015 radians, and 11.398 Hz, respectively, for the tumor. The immunohistochemical staining image **(H)** (×200) reveals thatHIF-1α staining positive cells is more than 50% and the staining intensity is more than 2 points.

The original imaging data were transmitted to the GE ADW 4.6 workstation, where ESWAN data were processed using the integrated software (Function Tool, GE Healthcare) to generate amplitude, phase, and R_2_^*^ maps. The DCE-MRI was analyzed and processed by the integrated software to acquire volume transfer constant (K^trans^), rate constant (K_ep_), and extravascular volume fraction (V_e_) maps. Two radiologists (Y.Z.X. and J.C.M., with 10 and 5 years of experience in uterine MRI, respectively) independently conducted the image analysis, oblivious to the specific pathological results. With T_2_WI and DWI images as reference, the parenchymal portion of tumor enhancement on the largest diameter slice on DCE-MRI was delineated as the region of interest (ROI). The parenchymal portion of the tumor should be included as much as possible (**Figures 2**, **3**), with an area larger than 0.5 cm^2^ and a distance greater than 0.2 cm from the tumor edge, and with exclusion of cystic lesions, bleeding, or adjacent vessels. Each observer draw the ROI for two times and recorded the mean value for all ESWAN and DCE-MRI parameters.

### HIF-1α immunohistochemistry

Immunohistochemical staining of HIF-1α was performed on 5-μm formalin-fixed paraffin-embedded tissue sections. The scoring of HIF-1α immunohistochemical results was based on the proportion of cells showing positive staining: 0 points, 0-25% (no staining or weakly positive); 1 point, 25-50% (moderately positive); 2 points, 50-75% (distinct positive); 3 points, 75-100% (strongly positive); and 4 points, 100% (entirely positive). Cases with scores 2 to 4 were categorized as the high-expression group, while the remaining cases were classified as the low-expression group (10, 15).

### Statistical analysis

Statistical analysis was performed with statistical software packages (SPSS 22.0 Chicago, IL, USA) and Med Calc 15.2.2 software (Med Calc Software, Ostend, Belgium). The interclass correlation coefficient (ICC) was used to evaluate the agreement of the measurement data between two observers. Data were determined to be generally distributed according to the Kolmogorov-Smirnov test and were compared using the independent samples t-test or the Mann-Whitney U test. General clinical information of the two groups of patients were defined as the number of cases or rate (%), and the comparison between groups was made using the chi-square test or Fisher’s exact probability method. Binary logistic regression was used to identify independent risk factors in predicting HIF-1α expression in EC. Receiver operating characteristic (ROC) analysis was used to evaluate the efficacy of different imaging parameters and their combination in predicting HIF-1α expression in EC, with the area under the curve (AUC), threshold, sensitivity, and specificity calculated. The Delong test was used to compare the variability among the AUC values. *P* < 0.05 was recognized as statistically significant.

## Results

### Patient’s characteristics

There were no notable variations between the two groups in clinical characteristics such as age, International Federation of Gynecology and Obstetrics (FIGO) stage, menopausal status, abnormal uterine bleeding, and type of pathology (*P* > 0.05) (as shown in **Table 2**).

**Table 2:** Comparison of clinicopathological parameters according to tumor HIF-1α expression level.

General Information	High-expression group (n = 65)	Low-expression group (n = 57)	X^2^/t	*P*-value
Age, years	58.45 ± 9.67	58.6 ± 11.37	-0.079	0.937
FIGO stage (no.)				
I, n (%)	50 (76.92)	43 (75.44)	2.591	0.490
II, n (%)	7 (10.7)	5 (8.77)		
III, n (%)	6 (9.23)	9 (15.79)		
IV, n (%)	2 (3.08)	0 (0.00)		
Menstrual status(no.)				
Before, n (%)	19 (29.23)	24 (42.11)	2.206	0.184
After, n (%)	46 (70.77)	33 (57.89)		
Abnormal uterine bleeding				
Yes, n (%)	26 (40.00)	27 (47.37)	0.671	0.466
No, n (%)	39 (60.00)	30 (52.63)		
Pathologictypes(no.)				
I	43 (66.15)	44 (77.19)	1.809	0.229
II	22(33.85)	13 (22.81)		

### Comparison of ESWAN and DCE-MRI parameters between two patient groups

**Figure 4:**
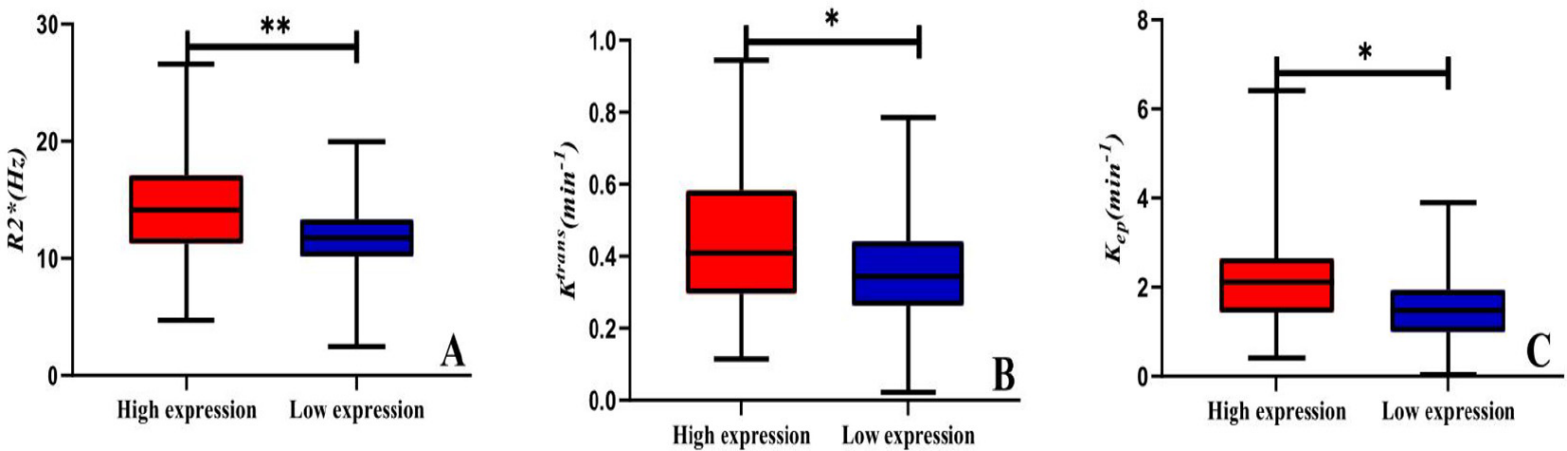
Box plots of parameters in the two patient groups. Comparison of R2^*^ **(A)**, K^trans^ **(B)**, and K_ep_ **(C)** between the two groups. *: P < 0.05, **: P < 0.01.

Data measurements by the two observers had a high consistency (ICC>0.75), and the mean of the two measurements were taken for further study (as shown in **Table 3**). The high-expression group had a significantly higher R_2_^*^, K^trans^, and K_ep_ values than the low-expression group (14.59 ± 4.06 *vs*. 11.99 ± 2.84 Hz, 0.45 ± 0.18 *vs*. 0.36 ± 0.14/min, and 2.17 ± 1.10 *vs*. 1.54 ± 0.80/min) (*P*<0.001, *P* =0.011, and *P* =0.001). However, no significant between-group difference was found concerning amplitude, phase, and V_e_ values (as shown in **Table 4** and **Figure 4**).

**Table 3:** Consistency of the imaging parameter measurements by the two observers.

Parameters	Patient group	Observer 1	Observer 2	ICC
R_2_^*^(Hz)	High-expression	14.73 ± 4.63	14.55 ± 4.14	0.831
	Low-expression	12.30 ± 3.33	11.70 ± 3.20	0.778
Amplitude	High-expression	808.22(628.09, 1054.78)	806.92(666.26, 1076.85)	0.975
	Low-expression	808.10(607.92, 1036.85)	798.03(634.44, 1068.60)	0.932
Phase(radians)	High-expression	0.03(0.01, 0.05)	0.03 ± 0.03	0.755
	Low-expression	0.04 ± 0.04	0.03 ± 0.05	0.822
K^trans^ (min^-1^)	High-expression	0.41(0.30, 0.60)	0.40(0.31, 0.59)	0.919
	Low-expression	0.35 ± 0.14	0.37 ± 0.16	0.906
K_ep_ (min^-1^)	High-expression	2.12 ± 1.14	2.02(1.37, 2.77)	0.896
	Low-expression	1.51 ± 0.79	1.54(0.98, 1.95)	0.895
V_e_	High-expression	0.24(0.16, 0.39)	0.24(0.14, 0.37)	0.848
	Low-expression	0.25(0.19, 0.44)	0.25(0.16, 0.40)	0.946

ICC, interclass correlation coefficient.

**Table 4:** ESWAN and DCE-MRI parameters measured for the two patient groups.

Parameters	High-expression(n=65)	Low-expression(n=57)	t/z	*P*
R_2_^*^(Hz)	14.59 ± 4.06	11.99 ± 2.84	3.738	<0.001
Amplitude	862.95 ± 285.66	838.80 ± 303.05	0.382	0.702
Phase (radians)	0.03 ± 0.03	0.04 ± 0.03	-0.544	0.586
K^trans^ (min^-1^)	0.45 ± 0.18	0.36 ± 0.14	2.527	0.011
K_ep_ (min^-1^)	2.17 ± 1.10	1.54 ± 0.80	3.374	0.001
V_e_	0.31 ± 0.26	0.32 ± 0.22	-0.682	0.495

### Binary logistic regression analysis to find independent risk factors for HIF-1α expression in EC

The parameters R_2_^*^, K^trans^, and K_ep_ values with statistically significant differences (P< 0.05) between the two groups were included in logistic regression analysis. Although the univariate analysis showed that R_2_^*^, K^trans^, and K_ep_ values were all favorable for evaluating HIF-1α expression in EC, the multivariate analysis showed that only R_2_^*^ and K_ep_ values were independent risk factors for assessing HIF-1α expression in EC (as shown in **Table 5**).

**Table 5:** Univariate and multivariate analyses to assess independent risk factors of HIF-1α expression in EC.

Parameters	Univariate analysis		Multivariate analysis	
	OR (95%CI)	*P*	OR (95%CI)	*P*
R_2_^*^ (Hz)	1.002 (1.001 - 1.003)	<0.001	1.003 (1.001 - 1.004)	<0.001
K^trans^ (min^-1^)	1.035 (1.010 - 1.060)	0.005	1.028 (0.998 - 1.059)	0.073
K_ep_ (min^-1^)	1.007 (1.003 - 1.012)	0.001	1.008 (1.002 - 1.013)	0.008

CI, confidence interval.

### Diagnostic efficacy evaluation of the different imaging parameters in identifying HIF-1α expression

**Figure 5:**
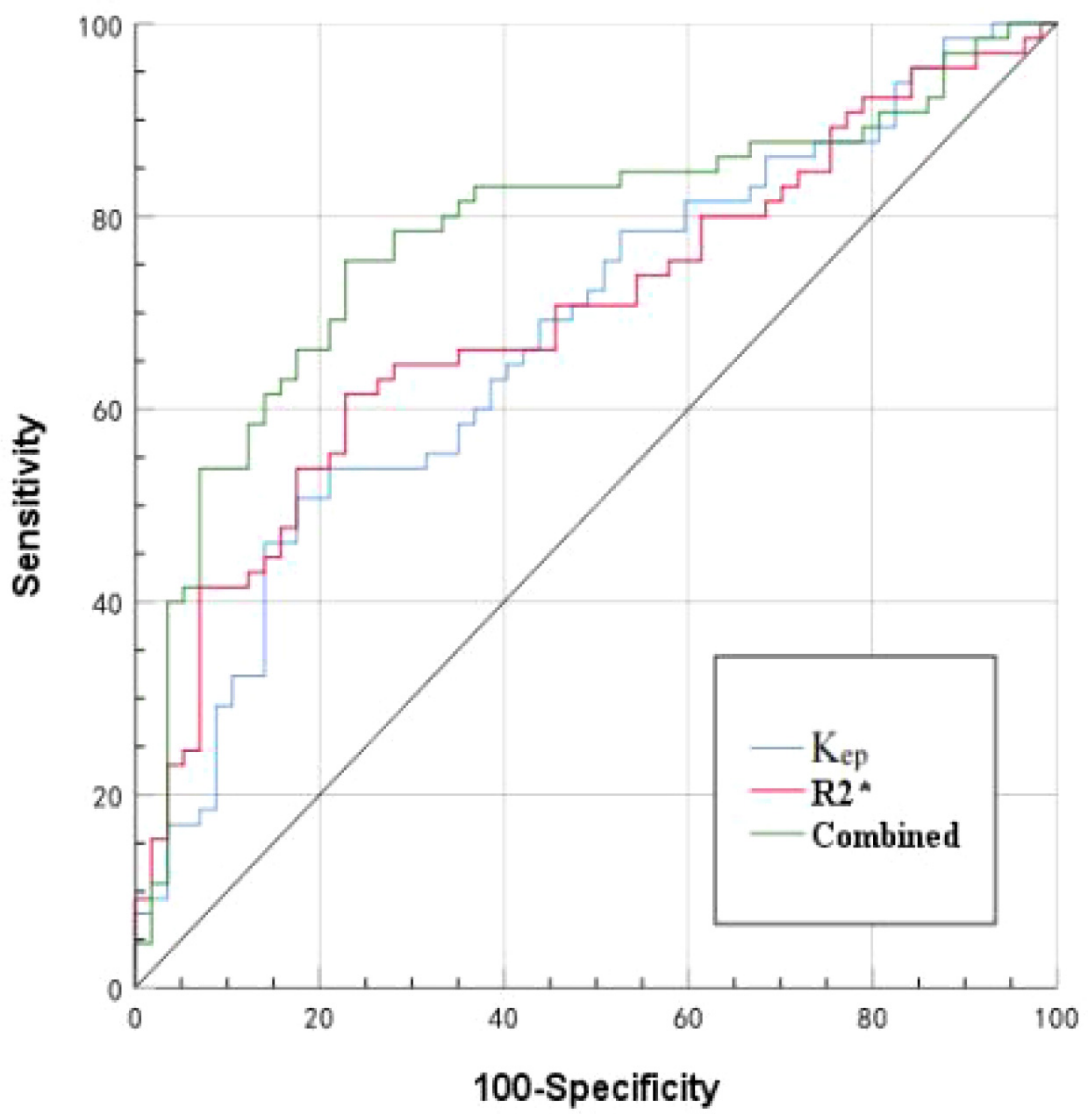
ROC curves of R_2_^*^, K_ep_, and R_2_^*^+K_ep_ to evaluate the expression status of HIF-1α in EC.

The AUCs, threshold, sensitivity, and specificity of R_2_^*^, K_ep_, and R_2_^*^+K_ep_ (R_2_* combined with K_ep_) were determined for prediction of HIF-1α expression in EC. The AUCs of R_2_^*^+K_ep_ were significantly higher than the AUCs of R_2_^*^ or K_ep_ (*P* < 0.05) (as shown in **Table 6** and **Figure 5**).

**Table 6:** Diagnostic efficacy of different imaging parameters for prediction of HIF-1α expression in EC.

Parameters	AUC (95%CI)	Threshold	Sensitivity (%)	Specificity (%)	Delong test results	
R_2_^*^ (Hz)	0.697 (0.603 - 0.790)	13.403	61.50	77.20	Z=2.412	*P*=0.016*
K_ep_ (min^-1^)	0.677 (0.583 – 0.772)	2.094	50.80	82.50	Z=2.431	*P*=0.015*
R_2_^*^+K_ep_	0.781 (0.696–0.866)	0.513	75.40	77.2	NA	NA

*AUC comparison between each parameter alone and their combination; NA, no comparison is needed.

## Discussion

HIF-1α is a crucial regulator of metabolic reprogramming in tumor cells. It governs the expression of genes related to hypoxia *in vivo*, including angiogenesis, erythropoiesis, glycolysis, cell adhesion, cell proliferation, and apoptosis (17), which can reflect the hypoxia microenvironment of tumors. Previous studies have shown that the hypoxia microenvironment accelerates the progression of solid tumors, increases invasiveness, enhances metastatic potential (18, 19). Over expression of HIF-1α will regulate the molecular signal pathway and mediate the expression of hypoxia-related genes, which will lead to insufficient arterial blood supply, low vascular density, low delivery efficiency of vascular tissue, changes in red blood cell flow, imbalance between oxygen supply and demand, and high invasiveness of tumor. Under hypoxia, chemotherapy drugs show low-level cytotoxicity (20, 21), reducing their therapeutic effect. According to the European Society of Gynecological Oncology (ESGO) treatment guidelines (22), EC with high prognostic risk factors should be treated with postoperative adjuvant therapy. Tumor type and grade are the criteria for risk stratification of tumors, which is more accurate when combined with molecular and immunohistochemical indicators (23, 24), and it has been demonstrated that HIF-1α is a high-risk factor for recurrence of EC (25). Thus, the accurate detection of HIF-1a expression in EC facilitates making individual treatment plans.

### Value of ESWAN combined with DCE-MRI in assessing HIF-1α expression in EC

ESWAN is a unique and sensitive imaging method for evaluation of oxygen content, calcium and iron deposits in tissues. R_2_^*^ (the transverse relaxation rate) value quantified by ESWAN, is directly correlated with the concentration of paramagnetic substances such as deoxyhemoglobin in tissues and is a sensitive index to evaluate the local oxygen content of tumors (26). It has been applied in the study of differential diagnosis of EC and endometrial polyp (27), expression of Ki-67 (28) and microsatellite instability in EC (29, 30). In this study, we found that the R_2_^*^ values of EC cases with high HIF-1α expression were higher than those with low HIF-1α expression. The reason may lies in that the high expression of HIF-1α can lead to increase in vascular endothelial growth factors (VEGFs) and other pro-angiogenic factors, and thus higher micro-vessel density. However, the weak and unstable vessel walls in tumor neovascularization can cause tumor tissue bleeding, blood stasis, excessive oxygen consumption, and increased paramagnetic substances such as deoxyhemoglobin and iron-containing hemoglobin (31), which can contribute to the increased R_2_^*^ values. Besides, higher HIF-1α expression is associated with more significant local tumor hypoxia and decreased blood oxygen content, which can lead to increase in paramagnetic substances like deoxyhemoglobin and iron-containing hemoglobin, resulting in elevated R_2_^*^ values. Meanwhile, R_2_^*^ has been identified as an independent risk factor for predicting HIF-1α over expression by multivariate analysis, indicating its potential and distinctiveness in assessing the anoxic microenvironment of tumor. For immobile tissues, the phase map shows the rotation angle of magnetization vector during relaxation, reflecting the difference in magnetic susceptibility between tissues by the changes in magnetic field. Increased paramagnetic material in organs will produce higher magnetic susceptibility. In our study, the phase values of EC patients in the high HIF-1α expression group tended to be lower than those of EC in the low expression group, but the difference was not statistically significant. One possible explanation is that high HIF-1α expression correlates with increased tumor malignancy (32). Tumors with higher malignancy have more local paramagnetic materials, causing a negative phase shift. This manifests as a low signal on the phase map, leading to a reduced phase value for the high HIF-1α expression group.

DCE-MRI sequences can quantitatively assess the contrast agent’s movement rate in and out of cells and blood vessels, thus obtaining information on tumor penetration and reflecting changes in the tumor microenvironment. Our findings demonstrated that the K^trans^ and K_ep_ values for the group with high HIF-1α expression exceeded those of the low expression group. K^trans^ represents the speed of the contrast agent’s movement from the vasculature to the extracellular space and the blood flow rate and vascular permeability, while K_ep_ represents the speed of contrast agent transfer from the extracellular space back into the vasculature, accurately reflecting capillary permeability (33). HIF-1α can instigate neoangiogenesis by activating angiogenic factors like VEGFs (34), leading to increased microangiogenesis in EC tumors. The blood vessels in tumors with higher expression of HIF-1α have disorganized endothelial cell arrangement and distorted morphology, resulting in increased permeability, enhanced contrast leakage, and easy reflux. Due to these factors, the K^trans^ and K_ep_ values in the high HIF-1α expression group were increased compared to the low expression group. Previous studies have found (35) that the K^trans^ and K_ep_ values of patients with soft tissue sarcoma in the high HIF-1α expression group were higher than those in the low expression group, which is consistent with this study.

Our multifactorial analysis revealed that both the R_2_^*^ and K_ep_ values can serve as independent risk factors for predicting high HIF-1α expression in EC. The expression level of HIF-1α can indicate the clinical biological characteristics of EC. R_2_^*^ value reflects the difference in contents of paramagnetic material and the dependence of blood oxygen level in the lesion, and K_ep_ value reflects the blood reflux velocity and capillary permeability in the lesion. High HIF-1α expression in the lesion increases blood flow rate and vascular permeability, leading to uncomplicated reflux of the contrast agent and a higher K_ep_ value. A weak and unstable capillary wall in the lesion can cause tumor bleeding and increased R_2_^*^ value. The combination of R_2_^*^ and K_ep_ integrates information from both image features, and thus significantly enhance the efficiency of evaluating high HIF-1α expression in EC.

### Limitations

Firstly, although the ROIs included the parenchymal portion of the tumor as much as possible, they still did not outline the tumor globally, and might miss some heterogeneous information.

Secondly, the ESWAN and DCE-MRI sequences were different in scanning orientation and layer thickness, which may result in slight mismatch among the ROIs of related parameters.

Thirdly, this study is a retrospective single-center study, and a multicenter prospective study is expected in the future for validation of current results.

## Conclusion

In conclusion, multimodal parameters by ESWAN and DCE-MRI were promise to serve as an important guide for quantitatively evaluating HIF-1α expression in endometrial cancer, which may help to predict the prognosis of the tumor and appropriately adjust the therapeutic regimen.

## Data Availability

The raw data supporting the conclusions of this article will be made available by the authors, without undue reservation.
